# Mature Nasopharyngeal Teratoma in a Child

**DOI:** 10.1155/2015/515474

**Published:** 2015-07-09

**Authors:** Ramesh Parajuli, Suman Thapa, Sushna Maharjan

**Affiliations:** ^1^Department of Otorhinolaryngology, Chitwan Medical College Teaching Hospital, P.O. Box 42, Bharatpur-10, Chitwan, Nepal; ^2^Department of Pathology, Chitwan Medical College Teaching Hospital, P.O. Box 42, Bharatpur-10, Chitwan, Nepal

## Abstract

Teratomas are neoplasms derived from the germ cell with components of all the three embryonic layers. These are rare neoplasms in head and neck region which can occur in any age group but are more prevalent in children. The present case is an 11-year-old girl who was brought with history of painless and progressive swelling in the oropharynx for 3 years with the associated left sided nasal blockage and nasal discharge. CT scan was suggestive of benign nasopharyngeal mass highly suspicious for lipoma. Excision of the mass was done under general anaesthesia. Peroperatively, it was a smooth, pedunculated mass arising from the left lateral wall in the nasopharynx. On cut section, it was solid to cystic mass similar to fatty tissue. Her HPE report came out to be mature teratoma of nasopharynx.

## 1. Introduction

Teratomas are congenital neoplasms derived from pluripotent cells from all the three germinal layers [1]. The incidence of teratoma is 1 in 40,000 live births which is relatively rare in the head and neck region, accounting for 2–5% of all paediatric teratomas [2]. Cervical region is the most common site followed by nasopharynx for the head and neck teratomas which usually grow slowly and progressively, pressing against adjacent structures [3]. Head and neck teratomas are rare and most of these are usually diagnosed during early childhood. While paediatric teratomas tend to be benign in nature, adult teratomas tend to be histologically and oncologically malignant [4].

The clinical presentation depends on the size and site of the lesion. The most frequent symptoms of head and neck teratomas include aerodigestive tract obstruction such as nasal blockage, difficulty in breathing and breast feeding, rhinorrhea, and snoring. Occasionally there can be history of cyanosis suggestive of respiratory obstruction and respiratory distress; similarly there may be associated dysphagia if the pedicle is long enough. The head and neck teratomas pose a unique challenge in the surgical management [5]. The aim of this paper is to present a first case of nasopharyngeal teratoma in Nepal.

## 2. Case Report

**Figure 1:**
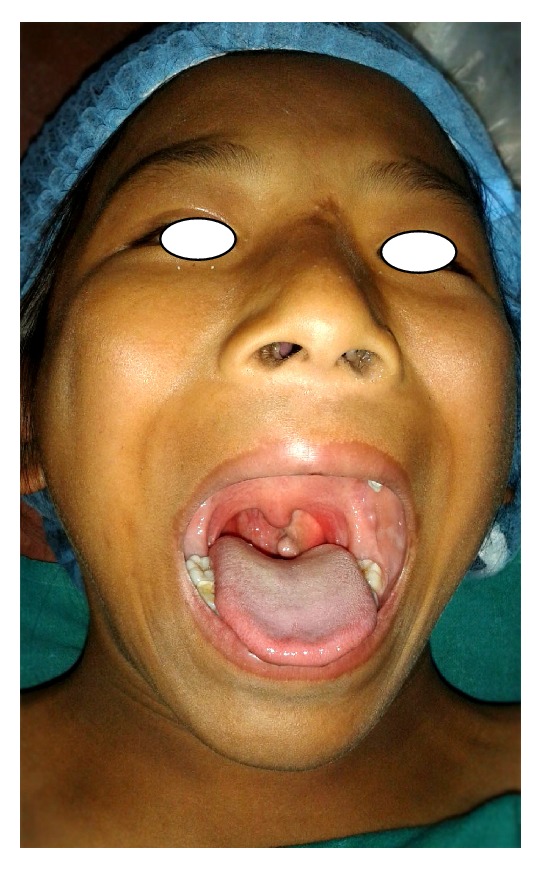
Preoperative photograph showing the mass hanging in the oropharynx.

An 11-year-old girl presented with the complaint of nasal blockage, recurrent nasal discharge, and open mouth breathing for 3 years. Recently her parents noticed an abnormal swelling inside mouth, which was insidious in onset, painless, and gradually progressive. There were no other ear and throat complaints. Physical examination revealed a smooth pinkish mass hanging in the oropharynx posterior to the left palatine tonsil (Figure 1). Flexible nasal endoscopy showed the pedunculated mass arising from left lateral wall of nasopharynx which seemed to be arising from just posterior to the Eustachian tube. The rest of the clinical examination was unremarkable.

**Figure 2:**
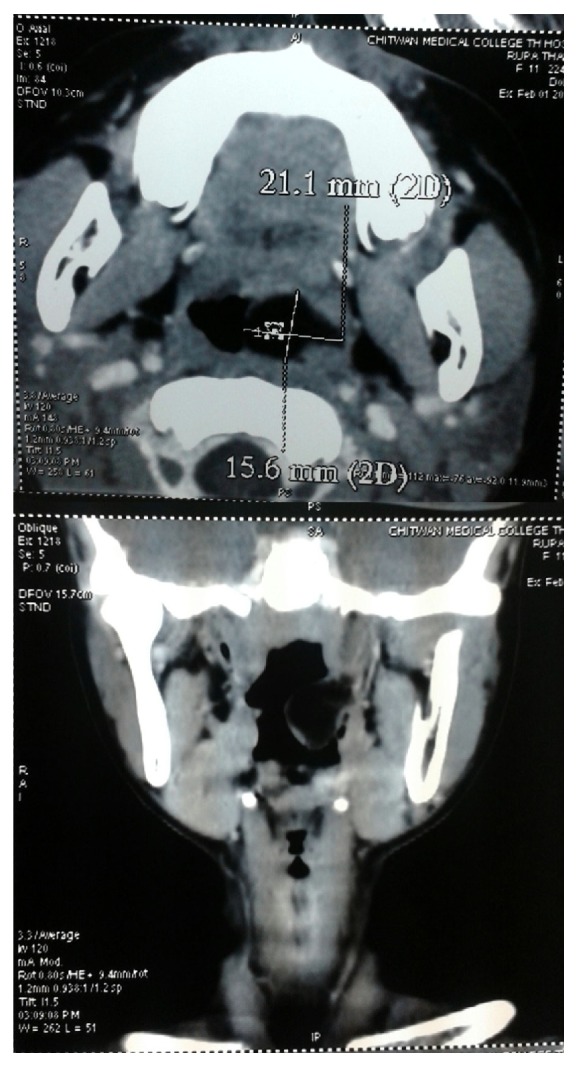
CT scan showing the soft tissue mass in the nasopharynx (nasopharyngeal mass) (axial and coronal view).

**Figure 3:**
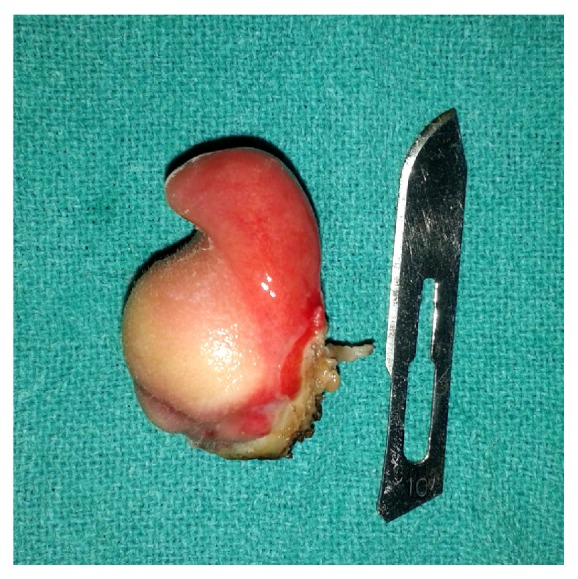
Excised (surgical) specimen.

**Figure 4:**
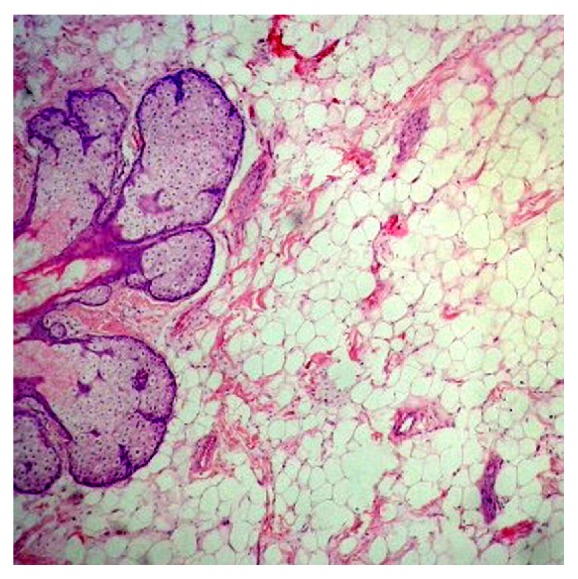
Histopathological examination showing keratinized stratified squamous epithelium with sebaceous and sweat glands and matured adipose tissue in the subepithelial stroma.

Computed tomography (CT) demonstrated a well circumscribed, heterogenous mass without intracranial extension originating from the nasopharynx suggestive of lipoma (Figure 2). The mass was excised with the help of bipolar electrocautery under general anaesthesia in tonsillectomy position using Boyle Davis mouth gag. Peroperatively it was a 3 × 2 cm single, smooth, pedunculated, well defined, firm mass having appearance of “newly hatched chicken” (Figure 3). The cut section of the swelling showed solid to cystic mass similar to fatty tissue. The specimen was sent for the histopathological examination (HPE) which showed keratinized stratified squamous epithelium with sebaceous and sweat glands and matured adipose tissue in the subepithelial stroma which was consistent with a mature teratoma (Figure 4). The postoperative period was uneventful. Postoperatively she was followed up for 3 months with no signs of recurrence.

## 3. Discussion

Teratomas are rare congenital neoplasms composed of elements from all three germinal layers. The teratomas are mostly found in the sacrococcygeal region and the head and neck region only accounts for 5% of all teratomas. In the head and neck region, the common sites of origin include the neck, oropharynx, nasopharynx, orbit, and paranasal sinuses [4, 6, 7]. They usually present at or soon after birth. Nasopharyngeal teratomas may be sessile or pedunculated. Most of the teratomas are diagnosed at birth with asphyxia neonatorum; however protruding mass from oral cavity and nares have also been reported [8]. They usually present with the features of upper aerodigestive tract obstruction such as nasal blockage, snoring, and rhinolalia. Complications of these neoplasms usually include the infection and pressure necrosis of the surrounding structures; rarely they may result into life threatening condition. Head and neck teratomas may be histologically mature or immature and oncologically benign. However teratomas may harbor malignant components and have the potential to exhibit an aggressive biological behavior. As a general rule, pediatric teratomas of the head and neck tend to be oncologically benign while adult teratomas tend to be histologically and oncologically malignant [4].

Teratomas are usually benign neoplasms which have been classified into 4 histological types such as dermoid, teratoid, true teratoma, and epignathi. Of these teratomas, dermoids comprise the vast majority; true teratomas occur less frequently. The exact etiology of these lesions is unknown. There are various theories regarding the origin of teratomas. For the head and neck teratomas, theory postulates that they arise from uncontrolled growth of a pluripotent cell originating in the region of the embryonic notochord [8].

Imaging investigations such as CT scan and MRI (magnetic resonance imaging) are useful to determine the site, size, and extent of lesions, to differentiate between solid and cystic mass, and to rule out the intracranial extension of the nasopharyngeal neoplasm, thus aiding in the surgical management [9]. In our patient, CT scan was performed which revealed a mass occupying the nasopharynx, with no signs of infiltration or intracranial extension.

Surgical excision is the treatment of choice for the mature nasopharyngeal teratoma, and the relapses are rare following complete excision of the mass [6]. In the present case, the nasopharyngeal teratoma was excised through transoral approach assisted with nasal endoscope; the lesion was removed from the nasopharyngeal region close to the pharyngeal ostium of the Eustachian tube using bipolar electrocautery. Although the complete extirpation is the ultimate goal of the treatment for head and neck teratomas, the surgical excision may be laborious and incomplete because of the complexity of the lesion and its extension into the surrounding structures in the complex anatomical area like nasopharynx [8].

## 4. Conclusion

Nasopharyngeal teratoma is a rare neoplasm which usually presents at or soon after birth with features of upper aerodigestive obstruction. The treatment of choice for mature teratoma is complete surgical excision. We present a first case of nasopharyngeal teratoma in child in Nepal along with the review of relevant literature.

## References

[1] Barksdale E. M., Obokhare I. (2009). Teratomas in infants and children. *Current Opinion in Pediatrics*.

[2] Shah F. A., Raghuram K., Suriyakumar G., Dave A. N., Patel V. B. (2002). Congenital teratoma of nasopharynx. *Indian Journal of Radiology and Imaging*.

[3] Chaudhry A. P., Lore J. M., Fisher J. E., Gambrino A. G. (1978). So-called hairy polyps or teratoid tumors of the nasopharynx. *Archives of Otolaryngology*.

[4] Cukurova I., Gumussoy M., Yaz A., Bayol U., Yigitbasi O. G. (2012). A benign teratoma presenting as an obstruction of the nasal cavity: a case report. *Journal of Medical Case Reports*.

[5] Zia-ul-Miraj Ahmad M., Brereton R. J., Madden N. P. (1996). Oronasopharyngeal teratomas. *Pediatric Surgery International*.

[6] Coppit G. L., Perkins J. A., Manning S. C. (2000). Nasopharyngeal teratomas and dermoids: a review of the literature and case series. *International Journal of Pediatric Otorhinolaryngology*.

[7] Ward R. F., April M. (1989). Teratomas of the head and neck. *Otolaryngologic Clinics of North America*.

[8] Dhingra K. K., Setia N., Khurana N. (2010). A rare case of congenital nasopharyngeal teratoma presenting with respiratory distress. *Indian Journal of Otolaryngology and Head and Neck Surgery*.

[9] Costa C. C., Guimarães V. D., Moura F. S., Chediack M. N., Fernandes E. J. (2014). Mature teratoma of the nasopharynx. *Brazilian Journal of Otorhinolaryngology*.

